# Future options of anti-angiogenic cancer therapy

**DOI:** 10.1186/s40880-016-0084-4

**Published:** 2016-02-15

**Authors:** Yihai Cao

**Affiliations:** Department of Microbiology, Tumor and Cell Biology, Karolinska Institute, 171 77 Stockholm, Sweden; Department of Medical and Health Sciences, Linköping University, 581 83 Linköping, Sweden; Department of Cardiovascular Sciences, University of Leicester and NIHR Leicester Cardiovascular Biomedical Research Unit, Glenfield Hospital, Leicester, LE3 9QP UK

**Keywords:** Angiogenesis, Cancer therapy, Anti-angiogenesis, Vascular endothelial growth factor, Biomarker

## Abstract

In human patients, drugs that block tumor vessel growth are widely used to treat a variety of cancer types. Many rigorous phase 3 clinical trials have demonstrated significant survival benefits; however, the addition of an anti-angiogenic component to conventional therapeutic modalities has generally produced modest survival benefits for cancer patients. Currently, it is unclear why these clinically available drugs targeting the same angiogenic pathways produce dissimilar effects in preclinical models and human patients. In this article, we discuss possible mechanisms of various anti-angiogenic drugs and the future development of optimized treatment regimens.

## Background

**Fig. 1:**
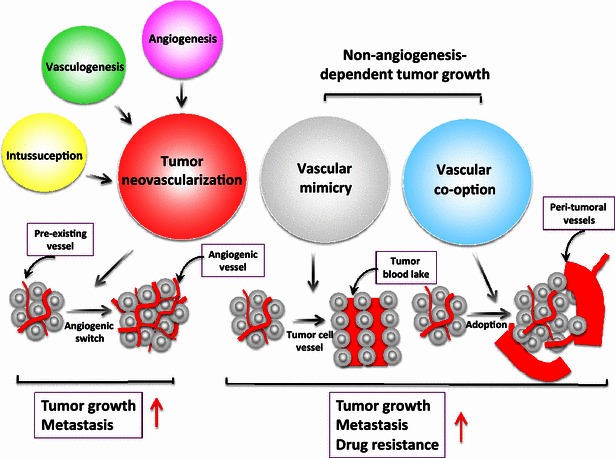
Mechanisms of tumor blood supply in supporting tumor growth, metastasis, and drug resistance. Angiogenesis, vasculogenesis, and intussusception contribute to tumor neovascularization and tumor growth. Tumors may also use alternative mechanisms, including vascular mimicry, by which tumor cells but not endothelial cells form vessel-like structures. These tumor cell-constituted vessel-like structures can be perfused with blood to form blood lakes that support tumor growth. Alternatively, tumor cells can also adopt pre-existing vasculatures in their surrounding tissues—a process called co-option—for growth and metastasis. It has been suggested that both vascular mimicry and co-option contribute to the development of anti-angiogenic drug resistance

Treating cancer by blocking tumor angiogenesis, which was proposed by Judah Folkman nearly 45 years ago [1, 2], is now a universally accepted mechanism. Decades of experimental evidence have shown that solid tumor growth is dependent on angiogenic formation of new blood vessels [3]. Therefore, blocking tumor angiogenesis could be a therapeutic option to treat all solid tumors. Indeed, in preclinical animal models, inhibition of tumor angiogenesis alone by agents that block angiogenic factors and by generic inhibitors produces robust anti-tumor activities [4]. Some of these generic inhibitors, such as angiostatin and endostatin, are present in humans (i.e., endogenous inhibitors, which may prevent the mature vasculature from further development [5–8]). Recent studies suggest that tumors can grow and invade through alternative mechanisms, including vascular mimicry and vascular co-option [9–14] (Fig. 1).

In preclinical tumor models, potent anti-cancer activity by angiogenesis inhibitors has been demonstrated; however, clinical studies with these inhibitors in human patients have shown different, and rather disappointing, data [15–17]. Targeting tumor blood vessels by angiogenesis inhibitors alone results in very few benefits for most cancer patients [15, 18–20]. Mechanistically, it is difficult to understand the differential responses of human cancer patients and mouse cancer models. Also, most clinically available anti-angiogenic drugs contain an anti-vascular endothelial growth factor (VEGF) component as the primary target, and tumors may produce non-VEGF angiogenic factors to induce angiogenesis [21]. Therefore, a small fraction of cancer patients may respond to anti-VEGF therapy, whereas other cancer patients might be intrinsically resistant to these drugs that do not specifically target the tumor angiogenic pathways. How do we discriminate responders from non-responders? Do we have many choices of drugs that target different angiogenic pathways? Would these drugs be given to patients for the rest of their lives? Currently, these important issues remain unresolved.

## Tumor size and patient survival

In almost all preclinical animal tumor models, the anti-tumor effect of angiogenesis inhibitors is assessed by suppression of tumor growth [4]. However, in clinical trials, survival improvement, especially improvement of overall survival, is the ultimate endpoint for clinical benefits. In deciding whether to approve new anti-cancer drugs, the United States Food and Drug Administration (USA FDA) uses survival improvement, not tumor size reduction, as the determining criterion. Does tumor size associate with patient survival? It is probably true for some cancer patients.

However, tumor size is not a reliable predictor of survival of most cancer patients, and large tumors may not necessarily mean shortened lifespan [22]. Of the most common causes of cancer-related death, metastasis is probably responsible for most mortality [23]. It is known that cancer invasion and metastasis can occur at the early stage of primary tumor development [24, 25]. In fact, in a substantial number of cancer patients, the first sign of malignant disease is metastasis; primary tumors are often not detectable [26]. This means that dissemination of malignant cells from primary sites occurs at the early stage of cancer development, probably when the primary tumor is at microscopic size [24, 25]. In support of this, in a zebrafish model, investigators found that cancer cell intravasation into the circulation occurred when a primary tumor had only a few hundred cells [24, 25]. In tumors, this small intravasation of tumor cells through the vessel wall occurs in surrounding pre-existing blood vessels, rather than in angiogenic vessels. Thus, when primary tumors lack an angiogenic phenotype, anti-angiogenic drugs would have only modest effects against cancer cell intravasation. Other primary causes of cancer-related death are cancer cachexia and other cancer-associated systemic diseases such as paraneoplastic syndrome [27, 28]. Cancer cells and cancer-associated inflammation are able to trigger a catabolic pathway that causes severe adipose and muscular atrophy [29]. Although the mechanisms underlying malignant cells in manipulating the macro environment and the metabolic pathway in cancer hosts, several inflammatory cytokines, including interleukin-6 and tumor necrosis factor-α, have been shown, in preclinical tumor models, to induce cancer cachexia [30, 31]. For most cancer patients with most cancer types, cancer cachexia is directly associated with shortened survival and poor quality of life. For example, patients with pancreatic cancer often develop cachexia, which is one of the main reasons for their poor survival prognosis [32].

Preclinical studies have commonly assessed the effect of any given anti-angiogenic agent on tumor growth for later clinical trials. Moreover, most studies aim to prevent tumor growth by simultaneously delivering drugs and tumor cells to host animals [4]. Established tumors are rarely treated with anti-angiogenic agents. In clinical settings, anti-angiogenic therapy is initiated during the late stage of tumor development [20], which is probably less dependent on angiogenesis. This illustrates how the currently available preclinical models are not fully relevant for human cancer patients. By better mimicking clinical situations, more reliable preclinical study results will be generated. Currently, such a clinically relevant model is still lacking. In clinical trials, most patients already have metastatic disease, and systemic delivery of anti-angiogenic drugs would inevitably affect metastatic tumor growth via blocking angiogenesis in metastatic nodules. This aspect is rarely considered in preclinical cancer models.

## Biologics- and small compound-based anti-angiogenic drugs

**Fig. 2:**
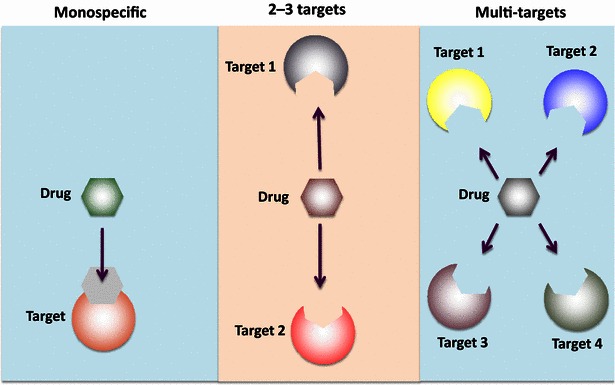
Anti-angiogenic drug targets. Monospecific bevacizumab, 2–3-targeted aflibercept and ramucirumab, and multi-targeted tyrosine kinase inhibitor anti-angiogenic drugs are currently used to treat cancer in human patients

**Fig. 3:**
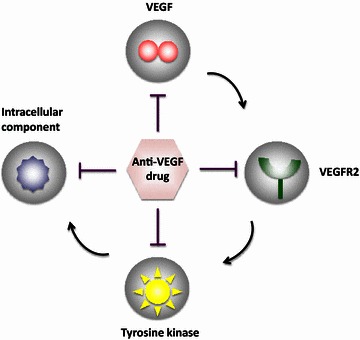
VEGF signaling and anti-VEGF drug targets. VEGF stimulates tumor angiogenesis by activating endothelial VEGFR2 and its downstream signaling. Drugs targeting various signaling components have been developed for clinical use. *VEGF* vascular endothelial growth factor, *VEGFR2* vascular endothelial growth factor receptor 2

Protein-based and chemical compound-based anti-angiogenic drugs are currently available for treatment of human cancers [21]. Although these drugs commonly target the VEGF signaling pathway (Fig. 2), they exhibit different specificities. The antibody-based drugs, including bevacizumab, aflibercept, and ramucirumab, are the most commonly used biologics, and they specifically bind to respective epitopes of the targeted molecules [33, 34]. Although these antibodies are monospecific with binding to their specific antigens, neutralization of a common target could potentially block functions of several angiogenic factors (Fig. 3). For example, ramucirumab binds to vascular endothelial growth factor receptor 2 (VEGFR2) and blocks its interactions with VEGF-A, VEGF-C, and VEGF-D. Similarly, soluble VEGFR-based drugs such as aflibercept can neutralize several ligands as one receptor binds to several ligands, including VEGF-A, VEGF-B, and placental growth factor [35]. Conversely, bevacizumab is a monospecific drug that blocks only VEGF-A without affecting other signaling pathways.

In contrast to antibody-based and soluble receptor-based biologics, small chemical compound-based drugs are far less specific. The most commonly used tyrosine kinase inhibitors (TKIs) that block VEGFR-mediated signaling pathways are small chemical molecules targeting a broad spectrum of kinases [36, 37]. Most VEGFR-TKIs, including sunitinib, sorafenib, and pazopanib, indistinguishably target VEGFR1, VEGFR2, and VEGFR3 signaling pathways. Additionally, these receptor inhibitors also block many other receptor kinases that are not parts of the VEGFR family but are often related to angiogenic signaling pathways, including members of the platelet-derived growth factor (PDGF) receptor and fibroblast growth factor (FGF) receptor families [38].

Theoretically, anti-angiogenic drugs that target abroad spectrum of signaling pathways would be more desirable and effective for treating cancer since malignant tissues are heterogeneous with different populations of tumor and host cells that produce various angiogenic factors. In this regard, anti-angiogenic TKIs would be more effective than antibody-based and soluble receptor-based drugs that solely target the VEGF pathway. However, clinical experience with anti-angiogenic therapy shows that TKIs may not necessarily be more effective than bevacizumab. Additionally, anti-angiogenic TKIs and bevacizumab show different profiles of toxicity, although both classes of drugs commonly cause some adverse effects. An important difference between biologics and TKIs is that antibody-based drugs have a longer half-life than small chemical molecules. They are inactivated using different metabolic pathways.

## Anti-angiogenic drug targets

Anti-angiogenic drugs target tumor blood vessels that exhibit heterogeneity [39]. However, none of available drugs are specifically delivered to the tumor tissue. They are delivered systemically to cancer patients, exposing all the tissues and organs to the drugs [22]. Would systemic delivery of anti-angiogenic drugs affect non-tumoral healthy vasculatures? In tumor-free healthy mice, systemic treatment with anti-angiogenic drugs, including an anti-VEGF neutralizing antibody and TKI-targeting VEGFRs, resulted in robust vascular regression in many tissues and organs. In all tissues, vasculatures in endocrine organs, including the thyroid, adrenal gland, ovary, and pancreatic β-islets, underwent robust regression in response to systemic anti-angiogenic therapy [40]. For example, after receiving only a 2-week treatment with a mouse version of bevacizumab, the mice lost more than 70% of pre-existing microvessels to the thyroid [41]. In addition to changes in vascular density, the endothelia underwent structural changes by replacing fenestrae with the intracellular vesiculo-vacuolar organelles. In normal physiological conditions, VEGF is a crucial hemostatic factor for endothelial cell survival and endothelium fenestrations in endocrine vasculatures. Thus, systemic inhibition of VEGF function would inevitably cause structural changes and decreases in vascular density. The anti-angiogenic drug-induced vascular changes also produce functional alterations in their respective organs. For example, thyroid hormones are significantly decreased after prolonged treatment with anti-VEGF drugs, resulting in hypothyroidism [41].

In addition to causing changes to the endocrine organs, anti-VEGF drugs also induce rigorous vascular regression in the liver, gastrointestinal wall, and kidney cortex [41]. Vascular regression inevitably creates a hypoxic environment in the targeted tissues and organs that eventually affects organ functions. These functional changes manifest as clinically adverse effects, such as hypertension, gastrointestinal perforation, hemorrhages, and protein in urine, which are commonly seen in cancer patients who are treated with anti-angiogenic drugs [15, 38, 42]. Paradoxically, off-tumor targets of anti-VEGF drugs can sometimes be beneficial for cancer patients [22]. This is particularly the case if circulating VEGF expression levels are extremely high in the patients whose tumors produce high amounts of VEGF. For example, in patients with von Hippel–Lindau (*Vhl*) gene-mutated renal cell carcinoma, VEGF expression levels can be very high [43]. Circulating VEGF also causes destructive effects in remote healthy tissues and organs, such as the bone marrow, liver, and spleen [44]. In this case, inhibition of VEGF-induced vascular impairment would potentially improve patient survival, as shown in preclinical models.

## Therapeutic timeline

**Fig. 4:**
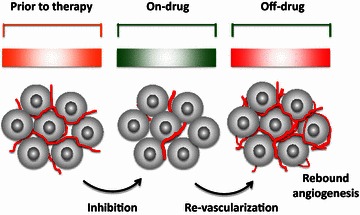
Effects of ON and OFF treatment with anti-angiogenic drugs on tumor vasculatures. Rapid revascularization and rebound angiogenesis can occur after treatment is discontinued

An important and clinically practical issue related to anti-angiogenic therapy is length of treatment. How long should a cancer patient be treated with anti-angiogenic drugs? What would happen if anti-angiogenic treatment was discontinued? Currently, no consensus exists regarding treatment timeline with anti-angiogenic drugs. As an anti-angiogenic component is added to the standard chemotherapy regimen, anti-angiogenic therapy follows the timeline of chemotherapy. In clinical practice, anti-angiogenic treatment will inevitably be discontinued. Additionally, anti-angiogenic treatment will likely result in adverse effects that make therapy withdrawal difficult. Similarly, if patients acquire drug resistance during treatment, this can also result in discontinuation of therapy. [A non-scientific reason for discontinuation of treatment is the economic burden incurred by patients (Fig. 4)]. In animal cancer models, discontinuation of anti-angiogenic therapy resulted in rapid regrowth of tumor blood vessels [45]. For small chemical compound-based drugs, revascularization occurs 2–3 days after drug withdrawal and reaches a maximal level around day 7. Around this time, revascularization generates a rebound time window that drives angiogenesis to a level higher than it was prior to treatment [41]. It is possible that rebound angiogenesis reflects the time course of angiogenic vessel growth before vascular remodeling and maturation.

It is unclear if, after discontinuation of anti-angiogenic therapy, rebound angiogenesis also occurs in human patients. However, reasonable speculation suggests that human tumors and mouse tumors would respond similarly. If rebound angiogenesis does occur in human cancer patients, discontinuation of anti-angiogenic therapy could potentially result in accelerated tumor growth. Thus, non-stop, lifetime anti-angiogenic treatment should be recommended. In support of this view, prolonged anti-angiogenic therapy has resulted in prolonged survival for human cancer patients.

## Drug resistance

Originally, researchers believed that angiogenesis inhibitors, especially the endogenous inhibitors such as angiostatin, endostatin, and other generic inhibitors, would not develop drug resistance of tumor cells because they target endothelial cells rather than tumor cells [46, 47]. Unlike malignant cells, endothelial cells, even those located in tumor tissues, have stable genomes and do not seem to use the canonical drug-resistant mechanisms. However, both experimental and clinical findings have challenged this view. Some studies showed that endothelial cells in angiogenic tumor vessels contain aberrant genomes that would not be present in healthy vasculatures [48]. It is unclear if the tumor-like aberrant genetic information in endothelial cells is transferred from tumor cells or if the intrinsic development genomic instability develops in endothelial cells. Inhibition of tumor angiogenesis could alter the cellular and molecular components in the tumor microenvironment, leading to development of drug resistance. For example, anti-angiogenic drug–induced vascular regression in the tumors creates tissue hypoxia in a local microenvironment, which augments expression levels of multiple angiogenic factors unrelated to the drug targets [36, 49]. Investigators have shown that anti-VEGF drugs develop resistance of tumor cells by this compensatory mechanism. Moreover, anti-VEGF drugs also tip the balance between various cellular compositions, including inflammatory cells and stromal fibroblasts, which are important sources of cytokines and non-VEGF angiogenic factors that contribute to drug resistance [50, 51].

Alternative mechanisms of tumor neovascularization that are not affected by drug targets also contribute to anti-angiogenic drug resistance. For example, vessel co-option, vascular mimicry, intussusception, and vasculogenesis support tumor growth and potentially inhibit anti-angiogenic treatment [10, 37, 52–54]. In patients who demonstrate intrinsic resistance to anti-VEGF therapy, non-VEGF angiogenic factors probably stimulate angiogenesis in their tumors. Thus, combination therapeutic approaches that target different angiogenesis signaling pathways would likely be more effective. Also, patients who take multi-targeted drugs such as TKIs would be less likely to develop drug resistance. Importantly, cross-communication between different angiogenic signaling pathways can generate synergistic effects, even though expression levels of each individual factor are low. For example, the synergistic effects between FGF receptor 2 and PDGF-BB on angiogenesis promote tumor growth and metastasis [55, 56].

## Mechanisms of combination therapy

In clinical practice, combination therapy represents a major mechanistic challenge [57]. Why would clinical benefits be achieved by combining anti-angiogenic drugs with chemotherapy? Why would anti-angiogenic treatment alone be sufficiently effective? A few possible hypotheses may explain the mechanism underlying combination therapy. One hypothesis suggests that treatment with anti-angiogenic drugs produces a normalized vascular phenotype, which increases vascular perfusion rather than decreases it [58]. In the presence of chemotherapeutic agents, increased vascular perfusion enables more cytotoxic drugs to reach the tumors, leading to increased tumor cell death. In other words, when administered with chemotherapeutics, anti-angiogenic treatment inhibits tumor growth. Also, the results of animal tumor models have demonstrated that anti-angiogenic drug-induced vascular normalization occurs within a limited time during treatment (i.e., the “vascular normalization window”) [59]. The mechanism underlying how combination therapy relates to vascular normalization is a paradox. If anti-angiogenic drugs induce vascular normalization and possibly blood perfusion in tumors, tumor growth would be accelerated. However, in both preclinical cancer models and clinical cancer patients, anti-angiogenic treatment does not promote tumor growth, although some researchers have suggested that the treatment facilitates cancer invasion [60, 61].

Another experimental study suggested that the mechanism underlying combination therapy can be explained by a decrease of chemotherapeutic toxicity [62]. Chemotherapeutics produce a broad spectrum of toxicity, including suppression of bone marrow hematopoiesis and high levels of circulating VEGF. Many cancer patients have high levels of circulating VEGF and manifest anemia [63]. A causal relationship between VEGF and anemia in human cancer patients has yet to be established, but studies of animal cancer models have shown that tumor-derived high-circulating VEGF causes severe anemia [44]. In high VEGF-producing tumor-bearing mice, chemotherapy and VEGF synergistically suppressed bone marrow hematopoiesis, resulting in early death [62]. Anti-VEGF treatment ablates VEGF-induced anemia and thus increases tolerance of chemotoxicity. Sequential delivery of anti-angiogenic therapy prior to the initiation of chemotherapy prolongs patient’s survival [62]. Anti-angiogenic drugs recover bone marrow hematopoiesis prior to chemotherapy and increase tolerance of chemotoxicity [62]. If this regimen were approved for at least a subset of human cancer patients, it would probably result in substantially increased survival benefits for these patients.

## Predictive biomarker-related issues

Mono-specific anti-VEGF drugs such as bevacizumab target only VEGF without binding to other proteins. VEGF expression levels would serve as a reliable predictive marker for selecting cancer patients who are likely to benefit from anti-VEGF therapy. Based on more than 10 years of clinical experience with various cancer types, simply measuring VEGF expression levels, in either the circulation or tumor biopsies, has not fulfilled the criterion for predicting responders [64–68]. Why would VEGF, as the sole target for bevacizumab, not serve as a reliable predictive marker for patient selection? There is no satisfactory answer to this puzzling question. However, some researchers have suggested that measuring different isoforms of VEGF might more reliably predict responders of anti-VEGF therapy [69–71]. Smaller VEGF isoforms, including VEGF121, lack heparin-binding affinity and diffuse distally from their productive sites. Additionally, proteolytically processed smaller versions of VEGF can also lack high heparin-binding affinity and can be transported to distal tissues and organs. Interestingly, these small versions of VEGF proteins have some predictive values, although their targets may not be limited to tumor tissues. It is possible that off-tumor targets of these small VEGF proteins predict their therapeutic values [22]. Indeed, based on preclinical and clinical findings, the potentially beneficial effects of anti-VEGF drug off-tumor targets have been proposed [22].

Many physiological, cellular, and molecular biomarker candidates related to anti-angiogenic therapy-induced adverse effects have been proposed, but in clinical practice physiological responses are the most commonly used biomarkers. For example, anti-angiogenic drug-induced hypertension has been associated with clinical benefits; however, the molecular mechanism underlying the benefit is unknown [72–78]. Given that adding anti-angiogenic components to conventional chemotherapy is widely used for the treatment of cancer, significant clinical benefits without selection biomarkers are truly valuable. Assuming a reliable predictive biomarker exists, treating a selected population of responders with anti-angiogenic drugs would likely markedly increase clinical benefits. Future efforts should focus on identifying such a reliable biomarker for clinical use.

## Adverse effects

Systemic delivery of anti-angiogenic drugs to cancer patients would inevitably expose non-cancerous healthy tissues to these drugs [40, 41]. In preclinical studies, investigators have shown that systemic treatment induces vascular changes in multiple tissues and organs. For example, in mice, systemic anti-angiogenic therapy caused marked regression of approximately 70% of microvessels in the thyroid and, to a lesser extent, in other endocrine organs, such as the adrenal gland and pancreatic islets [40, 41]. Additionally, anti-VEGF therapy caused a marked reduction in micro vasculatures in the liver, kidney, and gastrointestinal wall [40, 41]. Vascular changes in non-tumor tissues are associated with clinical adverse effects, including hypertension, hypothyroidism, gastrointestinal perforation, and cardiovascular disease [15, 79]. Since VEGF is an important hemostatic factor for maintaining the number and structure of microvessels in various tissues and organs, it is perhaps not surprising that anti-VEGF-based anti-angiogenic drugs would cause broad adverse effects.

How would anti-angiogenic drugs be directly delivered to tumorous tissues without affecting the healthy vasculature? Designing a new generation of targeted drugs would be a very challenging task. Even though anti-angiogenic drugs are locally injected into tumorous tissues, they still enter the circulation. Additionally, this approach would prevent anti-angiogenic agents from reaching metastatic tumors. In fact, clinical indications of using anti-angiogenic therapies approved by the U.S. FDA often include metastatic disease.

## Perspectives

Inhibition of angiogenesis for the treatment of cancer has been successfully translated into clinical use. The key issue is that patients who receive anti-angiogenic drugs experience relatively few clinical benefits. For patients with some cancer types, including pancreatic cancer and breast cancer, the addition of an anti-angiogenic component to chemotherapy has not produced meaningful improvement in overall survival. If all solid tumor growth depends on angiogenesis, why would anti-angiogenic treatments not be beneficial? Why would anti-angiogenic monotherapies fail to demonstrate clinical benefits? What is the mechanistic rationale of combination therapy with chemotherapeutics? Could a predictive marker be identified? How long should cancer patients be treated with anti-angiogenic drugs? What could happen if the anti-angiogenic therapy is discontinued? Would combinations of drugs that target different angiogenic pathways improve therapeutic outcomes? There are no unified opinions on these clinical issues. Possibly, an important means to address these issues is to establish clinically relevant cancer models in animals. Given sophisticated cancer biology, metastatic disease, and systemic disorders in cancer patients, the complex mechanisms underlying malignant disease cannot likely be simply explained. The same type of cancer in different patients may represent a different disease. Likewise, the same cancer in the same patient may represent a different disease at different stages of progression. This means that personalized medicine may not be sufficiently effective and that dynamic approaches should be developed for treating cancer at different stages during disease development. In clinical practice, developing both personalized therapy and dynamic therapy is an extremely challenging task.
